# Successful Transcatheter Arterial Radioembolization of Hepatocellular Carcinoma Via Left Internal Mammary Artery: A Case Report

**DOI:** 10.7759/cureus.22954

**Published:** 2022-03-08

**Authors:** Abdulmohsen Alhussaini, Shahbaz A Qazi, Yousof A AlZahrani

**Affiliations:** 1 Vascular and Interventional Radiology Unit, King Abdulaziz Medical City & King Abdullah Specialist Hospital, Ministry of National Guard Health Affairs, Riyadh, SAU

**Keywords:** collateral supply, extra-hepatic, loco regional, transarterial radioembolization, hepatocellular carcinoma (hcc)

## Abstract

Hepatocellular carcinoma (HCC) is the most common primary liver malignancy. Late-stage presentation, co-morbidities, and limited donor availability enable only a few patients to receive curative therapies. HCC is treated with liver resection and transplantation as the first line of treatment. Patients in advanced stages, on the other hand, are treated with a variety of locoregional treatments. Transarterial embolization (TAE), transarterial chemoembolization (TACE), and transarterial radioembolization (TARE) are different modalities of locoregional therapy for HCC with robust collateral circulation. One of the characteristics of HCC is that it is hypervascular, therefore, parasitic supply is not unusual. The left internal mammary artery (LIMA) is considered to be an uncommon parasitic supply in HCC. While TACE has been extensively reviewed in the literature, herein we report a successful case of TARE via the LIMA which was a safe and practical alternative for a patient with HCC.

## Introduction

Hepatocellular carcinoma (HCC), hepatocarcinoma, or hepatoma accounts for more than 90% of all cases of primary liver cancer [1]. HCC, which is the second leading cause of cancer-related deaths globally, has an incidence of 850,000 new cases per year [2]. Out of all primary liver cancers, HCC is the most common neoplasm [2]. Numerous risk factors for HCC development are identified, such as cirrhosis (chronic liver damage caused by fibrosis), hepatitis B virus (HBV) infection, hepatitis C virus (HCV) infection, alcohol abuse, and metabolic syndrome [2]. The vast majority of HCC patients are symptomatic (52.3%). Symptomatic cases tend to present commonly with abdominal pain, distension, and anorexia [3]. Late-stage presentation, co-morbidities, and limited donor availability enable only 20% of patients to receive curative therapies [4]. The first line of treatment for HCC is liver resection and liver transplant but patients who cannot undergo surgery or obtain a donated liver are good candidates for locoregional therapies which are used to manage patients with advanced HCC or as a bridging/downstaging therapy for early and intermediate disease [5]. Transarterial therapies consist of transarterial embolization (TAE), transarterial chemoembolization (TACE), and transarterial radioembolization (TARE) [6]. TARE, consists of intra-arterial delivery of radioactive material to the tumor, limiting systemic irradiation and preserving the healthy liver to a maximum extent. TARE appears to be a promising alternative to TACE [6]. Extrahepatic collaterals can be utilized for the loco-regional treatment of HCC by providing multiple access points for the tumor. According to Chung et al., the prevalence of extrahepatic collaterals is 17% in patients with HCC [7].

## Case presentation

**Figure 1:**
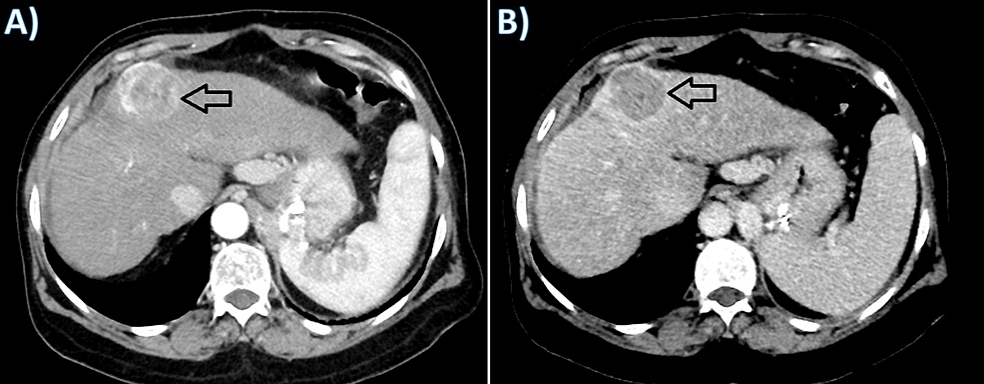
Axial contrast enhanced CT scan images. A: arterial phase and B: portovenous phase showing cirrhotic liver with hypervascular partially exophytic lesion in segment IV measuring 3.8 cm x 4 cm suggestive of HCC. HCC, hepatocellular carcinoma

A 57-year-old female who initially presented with abdominal pain was diagnosed with liver cirrhosis class B on Child-Pugh score due to non-alcoholic steatohepatitis and HCC in an outside hospital. CT showed cirrhotic liver with segment 4 subcapsular exophytic lesion measuring 3.8 cm x 4 cm compatible with HCC (Figure 1A). 

**Figure 2:**
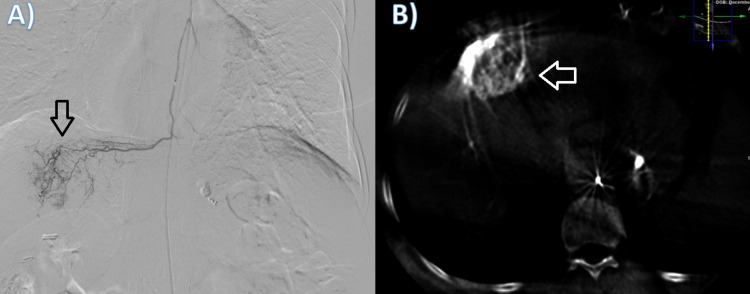
A: DSA of the left internal mammary artery showing tumoral blush supplied by distal branches. B: axial CBCT scan was obtained during the procedure confirming the tumoral supply by left internal mammary artery. DSA, digital subtraction angiography; CBCT, cone beam CT

The patient underwent TACE three times outside our institution hospital with no significant response. A decision was made to do TARE downstaging the tumor as a bridging therapy for liver transplantation. At first, hepatic artery mapping was performed to assess hepatopulmonary shunt and to evaluate anatomy. Multiple angiograms of celiac, superior mesenteric, gastroduodenal, right renal, left gastric, right internal mammary, and intercostal arteries showed that there is no supply to the lesion. No obvious supply for the tumor was identified. Therefore, the left internal mammary artery (LIMA) was cannulated using a vertebral catheter. An angiogram showed tumoral blush and a clear supply of the tumor from the LIMA crossing the midline (Figure 2A). A dynamic cone-beam CT (CBCT) was done and confirmed supply to the tumor as well (Figure 2B).

**Figure 3:**
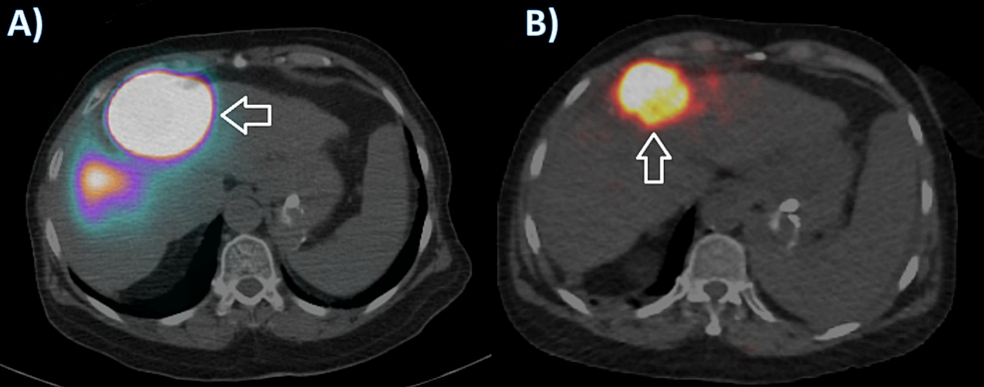
A: axial SPECT-CT image post technetium-99 macroaggregated albumin (Tc-99 MAA) injection confirming radiotracer localization within the known segment IV HCC. B: axial PET scan image following yttrium-90 (Y-90) infusion showing focal intense tracer localization in the known segment IV HCC. SPECT-CT, single-photon emission computed tomography-CT; HCC, hepatocellular carcinoma; PET, positron emission tomography

 This was followed by injection of technetium-99 macroaggregated albumin (Tc-99 MAA) via the LIMA distally. The hepatic artery mapping confirmed the LIMA supply of the HCC with MAA injection (Figure 3A). No extrahepatic radiotracer accumulation was found. A week later, radioembolization procedure was performed. After confirming the tumoral supply of the tumor, Y-90 SIR-sphere was injected as per the protocol (0.72 GBq) via the LIMA. Positron emission tomography (PET)/CT post therapy was done and showed focal intense tracer localization in the known segment 4 liver lesion with a minor activity in the left hepatic lobe which could be scattered counts (Figure 3B).

**Figure 4:**
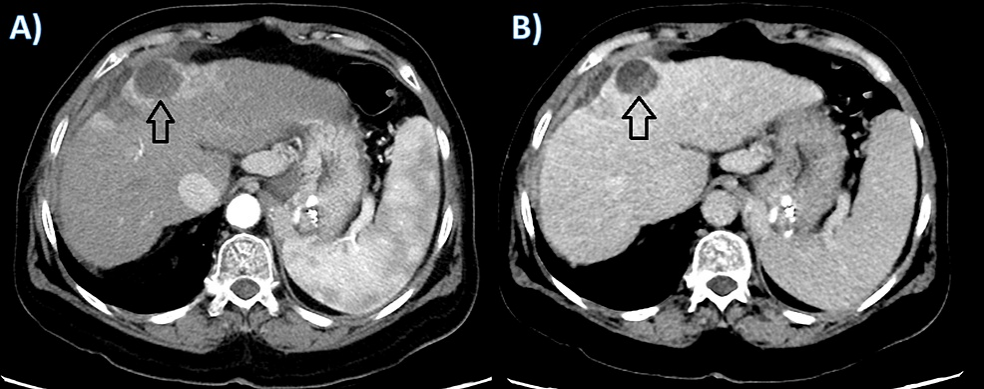
A: arterial phase; B: portovenous phase. A follow-up contrast enhanced axial CT scan image after three months in showing significant reduction in size to 2.4 cm x 2.5 cm and enhancement of the treated tumor in segment IV.

Three months later, a CT scan of the abdomen and pelvis was performed showing a significant response of the tumor with a reduction in size to 2.4 cm x 2.5 cm with post radioembolization changes (Figure 4). The patient's situation was discussed on the transplant and tumor board, and she was scheduled for a liver transplant.

## Discussion

According to Salem et al., the right inferior phrenic artery was the most prevalent collateral for HCC, accounting for 76.3% of the total collaterals detected in 1356 patients, followed by the right internal mammary artery, accounting for 6.9% [8]. Meanwhile, in the same study, the LIMA only accounted for 0.4% of the entire sample size. According to a second study by Fan et al., only 19 out of 3614 instances of HCC had a supply for the left internal mammary, accounting for barely 0.5% of the whole sample size [9]. Transarterial embolization (TAE), TACE, and TARE are examples of transarterial therapy for HCC. TARE involves delivering radioactive material to the tumor through the bloodstream, reducing systemic irradiation, and protecting the healthy liver to the greatest extent possible. TARE utilizes a variety of materials, including lipiodol, glass, and resin. Some radioisotopes have been used but only a few radionuclides are suitable for the treatment of tumors [6]. These radionuclides are 32P, 90Y, 131I, 166Ho, 177Lu, and 186/188Re, all of which are β-emitters. Only 90Y-loaded microspheres have been proven to be safe and effective in the treatment of cancers with acceptable toxicity [6]. Various techniques can be used to treat HCC with parasitic supply from the internal mammary artery. One of the treatment modalities prescribed in the literature is TAE. TAE is a procedure in which small particles made of tiny gelatin sponges or beads are injected. This blocks the artery and stops the flow of blood to the tumor or abnormal area of tissue. TACE is another modality for treating HCC with parasitic IMA involvement. TACE is a procedure that involves a transcatheter delivery of chemotherapy utilizing a lipiodol-based mixture plus an embolizing agent to accomplish strong cytotoxic and ischemic effects. According to Llovet et al., TACE outperforms TAE [10]. The results of the randomized clinical trial state that patients who underwent chemoembolization had a 15% less mortality rate than patients who underwent embolization of the selected patients with unresectable HCC. Kim et al. conducted a retrospective study to investigate the efficacy and feasibility of TACE via the internal mammary artery. They found that while the technical success rate of the chemoembolization was 55%, only three of the 97 patients were in complete remission [11]. According to Salem et al., TARE appears to be a promising alternative to TACE [8]. Patients with HCC treated with chemoembolization or radioembolization with Yttrium-90 microspheres had equal survival periods but radioembolization had a longer time-to-progression and less toxicity than chemoembolization.

## Conclusions

Transarterial radioembolization is a safe minimally invasive procedure that can be offered as an alternative treatment for patients with HCC. TARE also showed similar survival periods with less toxicity than other treatments. We report that TARE via the LIMA is a safe and feasible alternative for HCC with LIMA dominant collateral supply.
